# Evidence on implementing WHO Package of Essential Non-communicable (PEN) Diseases Interventions: a systematic review protocol

**DOI:** 10.1136/bmjopen-2025-112469

**Published:** 2026-02-19

**Authors:** Hongyi Xu, Alarcos Cieza, Enxhi Qama, Yiming Hu, Maoshu Li, Jing Yang, Jing Wu

**Affiliations:** 1 Department of Noncommunicable Diseases and Mental Health, World Health Organization, Geneva, Switzerland; 2 Swiss Paraplegic Research, Nottwil, Switzerland; 3 National Center for Chronic and Noncommunicable Disease Control and Prevention, Chinese Center for Disease Control and Prevention, Beijing, China

**Keywords:** health services, health services administration & management, primary health care, chronic disease

## Abstract

**Abstract:**

**Introduction:**

The WHO Package of Essential Non-communicable Diseases Interventions (WHO PEN) provides a core set of measures to prevent, detect and manage non-communicable diseases (NCDs) in low-resource settings. Many countries have adopted WHO PEN to strengthen primary healthcare, yet there is limited consolidated evidence on what components have been implemented and how WHO PEN has been implemented across different contexts. Understanding both the ‘what’ (disease modules, intervention activities, tools) and the ‘how’ (strategies, approaches, target populations and contextual factors) is crucial to assess the short-term to medium-term effects on health system readiness, provider performance, patient outcomes and long-term population health impact.

This protocol outlines a systematic review that will be updated as new evidence emerges and additional countries adopt or adapt WHO PEN. It represents the first systematic review focused on the implementation of the multifaceted interventions under WHO PEN. Findings will support efforts to sustain and scale up NCD interventions at the primary healthcare level and inform future updates of WHO PEN and related WHO guidance.

**Methods and analysis:**

We will search PubMed, Web of Science, Cochrane Library and Google Scholar for studies published up to June 2025, supplemented by grey literature and reference checking.

The review will follow the Preferred Reporting Items for Systematic Review and Meta-Analysis Protocols guidelines. Given the complexity, the review will be conducted in two stages. Stage 1 consists of an overview of review, mapping of existing review and evidence and guiding deeper inquiry of stage 2. Stage 2 will conduct a mixed-methods systematic review of the primary studies, forming the main output of this protocol.

**Ethics and dissemination:**

Ethics approval is not required. The protocol and findings will be disseminated through peer-reviewed publications, webinars and conferences.

**PROSPERO registration number:**

CRD420251064835.

**STRENGTHS AND LIMITATIONS OF THIS STUDY**

- The review uses a comprehensive protocol to guide stages of evidence synthesis.
- Multiple databases and grey literature sources will be searched to ensure broad and in-depth evidence coverage on implementation strategies.
- Both qualitative and quantitative data will be analysed using mixed-methods synthesis approaches.
- Variability in settings, study designs and reporting may limit the comparability and pooling of findings.
- The identification of the WHO Package of Essential Non-communicable Diseases Interventions (WHO PEN)-aligned activities may be constrained when studies do not explicitly label them as WHO PEN or report outcomes that could be influenced by overlapping interventions.

## Introduction

 Non-communicable diseases (NCDs) are responsible for the majority of global deaths annually and also cause significant morbidity, including disability. Despite some progress, the global target of reducing premature mortality from NCDs by one-third has not been met.^1^ More than two-thirds (approximately 85%) of premature mortality occurred in low- and middle-income countries (LMICs), which face challenges such as resource constraints, limited technical capacity and competing priorities.

The Global Action Plan for the Control of NCD 2013–2020 recommended that countries strengthen their health systems and address NCDs through people-centred primary healthcare (PHC) and universal health coverage (UHC). This includes the implementation of the WHO Package of Essential Non-communicable Disease Interventions (WHO PEN), strategically addressing NCDs by providing cost-effective interventions through primary healthcare and a health system strengthening approach.^1^ The commitment towards the integration of NCDs into primary healthcare has been advanced through country-level implementation of this package and its multifaceted interventions.^2^

### Package of Essential Non-communicable Disease Interventions

WHO PEN was considered a minimum standard and a starting point for action to address NCDs in primary care in low-resource settings, strategically prioritising prevention, strengthening early detection and timely treatment.^3^ First published in 2010 and updated in 2017 and 2020, WHO PEN 2010 features simplified clinical protocols on management of hypertension and diabetes, and interventions to prevent cardiovascular disease (CVD) using the total cardiovascular risk assessment.^4^

To facilitate the implementation, WHO PEN 2020^3^ provided various tools, including protocols for diagnosis and treatment of common NCDs, updated CVD risk prediction charts, a core list of medicines and technologies and technical packages (such as the HEARTS packages released in 2019–2020 for managing hypertension and CVD risk factors). The core elements of WHO PEN 2020 include modules for managing common NCDs, such as hypertension, diabetes, asthma and COPD, as well as early cancer diagnosis, lifestyle counselling, self-care and palliative care.

WHO PEN is used to integrate NCDs at primary care settings with resource constraints, as its components are designed to be delivered by primary care physicians and non-physician health workers. It leverages the global momentum towards UHC to include the essential NCD interventions in benefit packages for UHC. WHO PEN can also be adapted for use at humanitarian and postdisaster settings.^3 5^ Additionally, WHO PEN is recommended to guide joint prevention and control activities among patients with HIV in settings with high HIV prevalence.^6 7^

### Implementation of WHO PEN and context of the review

More than 40 countries, across all WHO regions, have embarked on the journey of strengthening health systems and supporting primary healthcare for addressing NCDs through implementing WHO PEN. WHO, including its regional and country offices and international experts from the collaborating centres, has provided workshops, technical support and implementation research protocols to assist with the implementation of PEN.^5 8 9^ A stepwise approach is recommended for implementing WHO PEN, including advocacy and stakeholders engagement, identifying pilots or demonstration sites, assessing primary health facility capacity and NCD services, developing service delivery package according to PEN, capacity building, evaluation and review and costing and scale-up plans.

However, since the package entails a range of multifaceted interventions, and the capacity and context of countries vary, the scope and stage of the implementation differ. For example, in the WHO African Region, adapting and using WHO PEN is a key target for countries.^8^ In the WHO Eastern Mediterranean Region, NCD service emergency kits have been developed for the NCD care in emergencies, adapted from WHO PEN.^6^ In the Philippines, after Typhoon Haiyan, WHO PEN was used to redevelop health services for NCDs. In the WHO Europe region, WHO PEN has been implemented to improve the quality of NCD care at health clinics.^10^

There are published systematic reviews on the implementation of WHO PEN (table 1). These reviews provide useful evidence on health system preparedness, factors and outcomes of implementation of WHO at the early stage. Some reviews focus on a specific geographic region or selected countries.^11 12^ The scope of these reviews includes health system readiness or public sector capacity,^12^ early outcome from pilot or feasibility studies^13^ and barriers or facilitators during implementation.^5^

**Table 1:** Checklist of WHO PEN core elements/tools

Tool ID	WHO PEN 2000 core elements/tools
1	CVD risk assessment
2	CVD chart
3	Management hypertension
4	Management diabetes II
5	Management asthma
6	Management COPD
7	Breast cancer
8	Cervical cancer
9	Healthy lifestyle counselling
10	Counselling on cessation of tobacco use
11	Self-care
12	Palliative care
13	Adapting PEN stepwise approach
14	Health facility assessment
15	Core list of medicine
16	Essential technologies and tools
17	Sample clinic record
18	Indicators

COPD, chronic obstructive pulmonary disease; CVD, cardiovascular disease; PEN, Package of Essential Non-communicable Diseases Interventions.

Overall, there is a lack of comprehensive understanding of the breadth of WHO PEN implementation. Some grey literature and country studies are not included. There is limited consolidated evidence on what components have been implemented and how WHO PEN has been implemented across different contexts. Understanding both the ‘what’ (disease modules, intervention activities, tools) and the ‘how’ (strategies, approaches, target populations and contextual factors) is crucial to assess the short-term to medium-term effects on health system readiness, provider performance, patient outcomes and long-term population health impact.

There are limited pooled analyses and subgroup analyses for the outcome, and the effectiveness of the implementation remains uncertain. Importantly, we need to determine whether the implementation so far has been effective and has had an impact on the population in need. It is equally important to distinguish between implementation failures and design flaws in the WHO PEN products.

Additionally, it is necessary to synthesise and appraise evidence on WHO PEN and its implementation strategies continuously and make it available to researchers and decision makers in countries to improve implementation.^14^

This review aims to examine and update evidence comprehensively on the implementation and effectiveness of WHO PEN. These will enhance the knowledge and practice of WHO PEN implementation and use, highlight ways to improve WHO PEN and implementation of WHO guidelines for greater impacts.

The specific objectives of this systematic review are as follows:

Objective 1: How has WHO PEN been implemented, and what specific components, approaches and delivery strategies were employed?

Objective 2: What outcomes and impacts arise from the implementation of WHO PEN?

Specific questions for objective 1 are as follows:

1. In what settings has WHO PEN been implemented (eg, rural or urban areas, socio-economic contexts, humanitarian settings, type of primary healthcare facilities)?
2. What is the scope of the implementation, and which core elements of WHO PEN (such as module, tool or protocol) have been used?
3. What interventions or disease areas are prioritised within each implementation?
4. How are high-risk populations or key populations defined?
5. What approaches have been used to implement WHO PEN in different settings?
6. What implementation strategies have been deployed to support WHO PEN delivery?
7. To what extent does WHO PEN implementation scale up, including expansion to national level?
8. What approaches have been used to sustain and scale up the implementation?
9. What barriers and facilitators influence the implementation of WHO PEN?

Specific questions for objective 2 are as follows:

1. What outcomes or indicators are used to measure the progress of the implementation?
2. What are the impacts of WHO PEN implementation on primary healthcare performance, including equity, efficiency and service delivery?
3. What are the impacts of implementation on population-level NCD prevention and control, including morbidity, mortality and critical CVD events?
4. What are the impacts of implementation on the healthcare workforce, including workload, roles and capacity?
5. Were any relevant inducive policies, including but not limited to universal access and/or equity, introduced during the implementation period?
6. What outcomes have been reported from long-term follow-up or scale-up efforts?
7. What economic evaluations are conducted and reported?

## Method and analysis

This systematic review protocol is registered at PROSPERO, the international prospective register of systematic reviews (https://www.crd.york.ac.uk/PROSPERO/), registration number: CRD420251064835. We report the methods according to the Preferred Reporting Items for Systematic Review and Meta-Analysis Protocols where possible.

Given the complexity of WHO PEN implementation across diverse contexts, the study will be conducted in two stages. Stage 1 consists of an umbrella review that synthesises existing systematic reviews on the implementation of WHO PEN or PEN-aligned interventions. This stage will help map the current evidence landscape and identify gaps, inconsistencies and areas requiring deeper investigation. Stage 2 will build directly on these insights by conducting a mixed-methods systematic review of primary studies, which constitutes the main output of this protocol. Narrative synthesis, thematic coding, established review frameworks and quantitative pooling will be incorporated where applicable during stage 2 of the project.

The scope and focus of stage 2 will be further refined based on the findings of stage 1, and any necessary methodological amendments will be documented and justified as the project progresses.

### Identifying relevant studies

#### Inclusion criteria for identifying relevant studies

To identify studies that provide evidence on the implementation of WHO PEN, predefined criteria will be applied.

#### General criteria

1. *Scope of implementation*: studies must demonstrate the implementation of WHO PEN.
2. *Publication date*: materials published since January 2010 up to June 2025 will be included.
3. *Type of materials*: both peer-reviewed literature and grey literature (eg, reports, theses, research) will be considered.

#### Specific characteristics of eligible studies

Studies must explicitly reference WHO PEN and be conducted in either research settings or routine health system environments. Studies include randomised controlled trials, quasi-experimental design, evaluation studies, implementation reports and other qualitative research describing implementation. Eligible studies may include, but are not limited to, the following:

- *Use or adaptation of WHO PEN tools including interventions*: studies that use or adapt WHO PEN tools (table 1) such as:
  - CVD risk charts;
  - HEARTS technical package;
  - disease management protocols;
  - WHO PEN standards.
- *Multicountry or benchmarking studies*: studies using WHO PEN as a benchmark or standard, including:
  - cross-sectional studies assessing health system or facility readiness;
  - studies on the prevalence and trends of NCDs and associated risk factors.
- *National adaptations*: implementation of national NCD intervention packages adapted from WHO PEN, such as IranPEN.
- *Integration into health services*: studies describing the integration of WHO PEN into existing health service delivery models.
- *Delivery of WHO PEN-recommended interventions*: studies involving:
  - task sharing or task shifting to non-physician health workers;
  - self-management strategies;
  - screening and innovative delivery strategies;
  - use of disease management algorithms;
  - diagnostic or referral criteria (eg, glucose measurement, body mass index, diabetes diagnosis, disease severity for referral).

#### Exclusion criteria

1. The study does not explicitly reference WHO PEN.
2. Studies such as opinion pieces, editorials, study protocols and commentary articles are excluded.

To reduce ambiguity and maintain focus, we will exclude studies that describe the integration of NCD interventions at primary healthcare facilities if they do not explicitly reference WHO PEN, even if they appear to align with its principles.

We will not exclude studies based on language, as we aim to capture all relevant evidence, including nuanced insights beyond selectively published materials.

Additionally, we will not limit inclusion to studies conducted in LMICs. For this review, we include all geographical areas.

### Search strategy

We will search the following databases for eligible articles published up to June 2025:

- PubMed;
- Web of Science;
- Cochrane Library;
- Embase;
- Google Scholar.

For grey literature—including reports, theses and research documents—we will search:

- the WHO website;
- WHO Institutional Repository for Information Sharing (WHO IRIS) and Pan American Health Organization Institutional Repository for Information Sharing (PAHO IRIS);
- NCD reports and relevant documents from WHO headquarters and regional offices;
- records from World Health Assembly and Regional Committee meetings;
- the WHO NCD Document Repository, particularly for national NCD clinical guidelines;
- public or government health department websites in countries known to have implemented WHO PEN.

We initially developed our search strategy using a combination of synonyms and Medical Subject Headings terms, including: “package of essential noncommunicable diseases”, “WHO PEN”, “noncommunicable diseases”, “integrated delivery system”, “primary health care”, “primary care” and “health center”.

However, after trial searches, we refined our strategy to use only the keywords “package of essential noncommunicable” or “WHO PEN”, as these yielded approximately 3400 relevant records, aligning more closely with our research objectives.

Details of the final search strategy are provided in online supplemental annex 1.

### Screening and selection

The search results will be imported into Endnote X8, where duplicates will be removed both automatically and manually.

- Initial screening of titles, abstracts and keywords will be conducted by reviewer HX.
- If abstracts are unavailable or unclear, or if the reference to WHO PEN is uncertain, reviewer HX will review the full text to determine whether the study meets the inclusion criteria.
- A 20% random sample of the screened records will be independently reviewed by EQ and YH. Any disagreements will be discussed with AC and resolved by consensus.
- Reviewers EQ, YH and ML will assist in identifying and reviewing grey literature.

If a study is considered potentially eligible based on the title and abstract but the full text is in a non-English language, we will use an AI translation tool to translate the full text into English. The study will be included if it meets the inclusion criteria after translation.

We will also contact experts and professional networks to request any relevant grey literature (eg, reports, conference proceedings) on the implementation of WHO PEN in their respective countries.

For the overview of the review, HX will screen and select all review or systematic reviews that explicitly reference WHO PEN and are directly relevant to the WHO PEN and its implementation. YH and ML screen citations of references to identify potentially relevant reviews by examining titles and abstracts.

### Data extraction and quality assessment

Reviewer HX will create a standard data extraction form. HX, EQ, YH and ML will extract data from included studies using the standardised form. The following information will be collected: first author, year of publication, country of study, setting and population, study type, sample size, study period and duration, scope of implementation (table 1), interventions and diseases addressed, implementation strategies, implementation stage, related policy, scale-up approaches, outcome measures, key findings and conclusion and good practice recommendations.

Reviewer YH, ML and EQ will independently validate 20% of the selected studies.

When feasible, we will label or stratify implementation settings and factors related to inequalities,^15^ such as place of residence, race/ethnicity/culture/language, occupation, sex, education, socioeconomic status, social capital and other vulnerability factors (eg, age, disability).

Based on the existing systematic review^13^ and WHO PEN and other indicator frameworks,^16^ we will extract outcome and impact measures in the categories mentioned in table 2.

**Table 2:** Outcome and impact measurements of WHO PEN implementation

Outcome category	Definition
PHC system	Strengthen PHC system_efficiency_reduction of hospitalisation due to hypertension diabetes
PHC system	Strengthen PHC system_quality of care including adherence to protocol, strengthen referral, service gaps
PHC system	Strengthen PHC system_equity
Health system general	Strengthen health system_empower middle-level health workforce and competency
Health system general	Strengthen health system_improve service utilisation of public facilities
Health system readiness	Workforce capacity, availability of core medicines, diagnostic and equipment
NCD and risk factors	Control NCD and risk factor_hypertension (blood pressure, blood pressure control)
NCD and risk factors	Control NCD and risk factor_diabetes (glucose, HbA1c, LDL-cholesterol)
NCD and risk factors	Control NCD and risk factor_asthma
NCD and risk factors	Control NCD and risk factor_prevalence of risk factors
NCD and risk factors	Control NCD and risk factor_smoking cessation
NCD and risk factors	Control NCD and risk factor_healthy lifestyle
NCD and risk factors	Control NCD and risk factor_CVD risk
Patients	Patients_satisfaction, retention in care, increase access to service
Patients	Patients_awareness and knowledge
Patients	Patients_medication adherence
Patients	Patients_out-of-pocket expense
Patients	Patients_quality of life, improved productivity
Health impact	Health impact_identify underdiagnosed patients
Health impact	Health impact_treatment coverage
Health impact	Health impact_mortality reduction
Health impact	Health impact_critical CVD events reduction
Economic outcomes	Cost, cost-effectiveness
Others	As reported in the included publications

CVD, cardiovascular disease; HbA1c, haemoglobin A1c; LDL, low-density lipoprotein; NCD, non-communicable disease; PEN, Package of Essential Non-communicable Diseases Interventions; PHC, primary healthcare.

For countries that have initiated national scale-up of WHO PEN, we will extract population-level data on treatment and control of hypertension and diabetes. These data will be sourced from included studies and supplemented with population-based surveys, particularly those with national or subnational biochemical measurements (eg, WHO STEPwise approach to NCD risk factor surveillance (STEPS)).

We will also seek data on prevention of acute events and complications, prolongation of stable periods for patients with NCD, reduction in diagnostic delays, equity and efficiency measures (eg, cost savings).

Two reviewers, YH and ML, will assess independently the quality included under objective 2 using Cochrane risk-of-bias tool^17^ for randomised trials and Critical Appraisal Skills Programme (CASP) checklists for other research studies. They will also assess bias and heterogeneity among similar studies (≥3 studies) using a random-effects model. An I² statistic >75% will be considered indicative of substantial heterogeneity.

For the reviews selected for the overview of review, HX will extract information related to location, study characteristics, relevance to WHO PEN implementation, quality assessment, review findings and conclusions.

### Data synthesis and analysis: qualitative and quantitative

We will employ a mixed-method approach to data synthesis, combining narrative synthesis, thematic coding, the use of existing review frameworks and quantitative pooling where appropriate.

To analyse barriers and facilitators to WHO PEN implementation, our method will be primarily inductive while also allowing for deductive coding guided by implementation science frameworks such as the Consolidated Framework for Implementation Research (CFIR). This blended approach will help ensure that the analysis captures both emergent insights and concepts already well described in the literature.

- Reviewer HX will conduct an open inductive coding process using NVivo software or Excel.
- Group-related codes into preliminary categories.
- Develop themes and subthemes by identifying patterns, relationships and overarching concepts, and map these to themes identified in existing systematic review^5 10^ (table 3).
- New or emerging themes and subthemes not captured in previous frameworks will be added as needed.
- Use the CFIR framework to classify qualitative findings, applying both deductive (framework-based) and inductive (data-driven) approaches.
- Reviewers AC and EQ will independently validate the coding of the thematic structure to ensure consistency and reliability.

**Table 3:** Themes and subthemes of facilitators and barriers to WHO PEN implementation

Themes	Subthemes
Health systems	Leadership/Governance
	Health financing
	Health workforce
	Medicine and technologies
	Information and research
	Service delivery
External factors	Knowledge and culture
	Economic and financing

PEN, Package of Essential Non-communicable Diseases Interventions.

We will also code and summarise any recommendations or users’ experience on WHO PEN products itself.

To guide interpretation, we will categorise studies into four implementation phases:

1. Assessment—for example, facility readiness assessments, needs identification and analysis of target population.
2. Feasibility—for example, testing key features of WHO PEN or delivery strategies in specific context.
3. Implementation—for example, piloting WHO PEN in multiple districts or subnational regions.
4. Evolution—for example, costing studies, resource allocation and national scale-up efforts.

We will apply this framework as an analytic pathway to gain deeper insights into the implementation process and outcomes across different stages and settings.

### Quantitative analysis

For objective 2, due to the anticipated heterogeneity across studies—including differences in target populations, intervention packages, study designs and outcome measures—a meta-analysis may not be feasible. Our PICO framework for this review is presented in table 4.

**Table 4:** PICO framework—primarily applied to objective 2

Population	Interventions (package)	Comparison	Outcomes
Adult	WHO PEN interventions (list as below):	Usual care	WHO PEN outcomes (see table 2 for details)
Patients with non-communicable disease	CVD risk assessment	Any comparator	Primary healthcare system
	CVD chart		Health system
	Management of hypertension		NCD control and risk factors
	Management of type 2 diabetes		Patients perception
	Management of asthma		Health impact
	Management of COPD		Economic outcomes
	Breast cancer		
	Cervical cancer		
	Healthy lifestyle counselling		
	Counselling on cessation of tobacco use		
	Self-care		
	Palliative care		

COPD, chronic obstructive pulmonary disease; CVD, cardiovascular disease; NCD, non-communicable disease; PEN, Package of Essential Non-communicable Diseases Interventions.

#### PICO framework

We will take the following steps into account:

- Refine the PICO framework including priority outcomes, based on insights from both the overview and the qualitative review.
- Consider subgroup analysis or data pooling if at least two studies—such as randomised controlled trials or quasi-experimental studies—report on the same outcome measures.
- Where applicable, define the comparison group as WHO PEN versus usual care or any other relevant comparator.
- Calculate risk ratios (eg, proportion of patients with controlled blood pressure or blood sugar), mean differences of continuous outcomes or changes from baseline, depending on the type of outcome (table 2).
- If data pooling is not feasible, present quantitative findings using tables and graphs to summarise key results.

When possible, we will assess the impact of WHO PEN implementation on health equity by conducting subgroup analyses across dimensions of health inequality (eg, socioeconomic status, gender, geography).

For the scale-up analysis, we will exclude studies categorised under the assessment and feasibility phases. We define sustained or long-term improvements as those observed over a period of 12 months or longer.

For the overview of the review, the data synthesis will be narrative, focusing on the scope of existing review, mapping evidence landscape and identifying gaps in the evidence base, understanding practices and existing efforts on examining effectiveness of WHO PEN implementation.

### Data management and team roles

- HX will lead data extraction, synthesis and analysis.
- EQ will assist with the qualitative synthesis.
- YH and ML will support quantitative data synthesis and statistical analysis, including pooled analysis and meta-analysis where appropriate.
- All authors will contribute to the validation and interpretation of the findings.

### Presentation of results

- Study selection will be illustrated using a Preferred Reporting Items for Systematic Reviews and Meta-Analyses flow diagram.
- Results will be presented in tables, graphs and narrative summaries, aligned with the review objectives and questions.
- HX will develop manuscripts for peer-review journal submission. All authors will contribute to the manuscript preparation and revision.
- We will compare our findings with existing systematic reviews on WHO PEN implementation or similar multifaceted NCD interventions at the primary healthcare level, even if they do not explicitly reference WHO PEN.
- Finally, we will highlight the unique contributions of this review, particularly in terms of new evidence to inform improvements in WHO PEN tools and implementation strategies.

### Planned updates to the systematic review

We will begin by producing an up-to-date baseline systematic review following the methods outlined in this protocol. Subsequently, updates will be conducted approximately every 3 years through the existing WHO Collaborating Centres and their network. These updates will follow the same protocol or a modified version, depending on the emergence of new evidence and evolving policy or practice needs. Ultimately, the updated evidence synthesis will inform future revision of WHO PEN and implementing other related guidelines.

## Ethics and dissemination

Ethics approval is not required since this is a systematic review with peer feedback from relevant stakeholders.

The protocol and study findings will inform the development of manuscripts for submission to peer-reviewed journals and will be disseminated through webinars and conferences.

This is the first systematic review of its kind to examine the implementation of multifaceted and complex interventions under WHO PEN. The findings will provide valuable insights for stakeholders aiming to sustain and scale up NCD interventions at the primary healthcare level. The review and planned updates of the evidence synthesis will offer timely evidence to support the update and application of WHO guidelines, including WHO PEN.

## Supplementary material

10.1136/bmjopen-2025-112469 online supplemental file 1

## References

[1] Secretary-General, U (2025). Progress on the prevention and control of non-communicable diseases and the promotion of mental health and well-being: report of the secretary-general. https://digitallibrary.un.org/record/4076846?ln=en&v=pdf.

[2] Nations., G.A.U (2025). Progress on the prevention and control of non-communicable diseases and the promotion of mental health and well-being. https://docs.un.org/en/A/79/762.

[3] World Health Organization (2020). WHO package of essential noncommunicable (PEN) disease interventions for primary health care.

[4] World Health Organization (2010). Package of essential noncommunicable (Pen) disease interventions for primary health care in low-resource settings.

[5] Aminpour M, Aryankhesal A, Hashjin AA (2024). Implementation of the Package of Essential Non-Communicable (PEN) Disease Interventions in Low-Resource Settings: A Systematic Review. Iran J Public Health.

[6] World Health Organization (2023). Integrating the prevention and control of noncommunicable diseases in HIV/AIDS, tuberculosis, and sexual and reproductive health programmes: implementation guidance.

[7] World Health Organization (2016). Consolidated guidelines on the use of antiretroviral drugs for treating and preventing HIV infection: recommendations for a public health approach.

[8] Tesema AG, Ajisegiri WS, Abimbola S (2020). How well are non-communicable disease services being integrated into primary health care in Africa: A review of progress against World Health Organization’s African regional targets. PLoS One.

[9] Luciani S, Nederveen L, Martinez R (2023). Noncommunicable diseases in the Americas: a review of the Pan American Health Organization’s 25-year program of work. Rev Panam Salud Publica.

[10] Muratalieva E, Nendaz M, Beran D (2022). Strategies to address non-communicable diseases in the Commonwealth of Independent States countries: a scoping review. Prim Health Care Res Dev.

[11] Albelbeisi AH, Albelbeisi A, El Bilbeisi AH (2021). Public Sector Capacity to Prevent and Control of Noncommunicable Diseases in Twelve Low- and Middle-Income Countries Based on WHO-PEN Standards: A Systematic Review. Health Serv Insights.

[12] Chitani S, Dubey DK (2023). Healthcare system readiness to implement world health organization package of essential non-communicable disease intervention in african low-middle income countries: a systematic review. Preprints.

[13] Tripathy JP, Mishra S (2021). How effective was implementation of the package of essential non-communicable disease (PEN) interventions: A review of evidence?. Diabetes Metab Syndr.

[14] Correia JC, Lachat S, Lagger G (2019). Interventions targeting hypertension and diabetes mellitus at community and primary healthcare level in low- and middle-income countries:a scoping review. BMC Public Health.

[15] Welch V, Dewidar O, Tanjong Ghogomu E (2022). How effects on health equity are assessed in systematic reviews of interventions. Cochrane Database Syst Rev.

[16] World Health Organization (2022). Noncommunicable disease facility-based monitoring guidance: framework, indicators and application.

[17] Higgins JPT, Altman DG, Gøtzsche PC (2011). The Cochrane Collaboration’s tool for assessing risk of bias in randomised trials. BMJ.

