# Supplementary material for: Evidence on implementing WHO Package of Essential Non-communicable (PEN) Diseases Interventions: a systematic review protocol
Source: BMJ Open. 2026 Feb 19;16(2):e112469. doi: 10.1136/bmjopen-2025-112469 (PMC12927286; doi:10.1136/bmjopen-2025-112469)
Supplement: online supplemental file 1 [file bmjopen-16-2-s001.docx]

**Annex 1. Searching strategy for Pubmed, Web of Science, Cochrane Library, and Google Scholar**

"package of essential noncommunicable" OR "package of essential non-communicable" OR “WHO PEN”
