# Supplementary material for: Evidence on implementing WHO Package of Essential Non-communicable (PEN) Diseases Interventions: a systematic review protocol
Source: BMJ Open. 2026 Feb 19;16(2):e112469. doi: 10.1136/bmjopen-2025-112469 (PMC12927286; doi:10.1136/bmjopen-2025-112469)
Supplement: Supplementary file 2 [file bmjopen-2025-112469.reviewer_comments.pdf]

PEER REVIEW HISTORY

BMJ Open publishes all reviews undertaken for accepted manuscripts. Reviewers are asked to complete a checklist review form and are provided with free text boxes to elaborate on their assessment. These free text comments are reproduced below.

ARTICLE DETAILS

Title (Provisional)

Evidence on Implementing WHO Package of Essential Noncommunicable (PEN) Diseases Interventions: A Systematic Review Protocol

Authors

Xu, Hongyi; Cieza , Alarcos; Qama, Enxhi; Hu, Yiming; Li, Maoshu; Yang, Jing; Wu, Jing

---

VERSION 1 - REVIEW

**Reviewer**	**1**
**Name**	**Shawon, Riffat Ara**
**Affiliation**	**University of Washington, Epidemiology**
**Date**	**23-Dec-2025**
**COI**	**None**

Dear Authors, thank you for taking on the task of compiling evidence on PEN intervention. Here are a few of my comments for your consideration.

- Abstract includes that this will be a "live" review. I am not clear from the abstract how it will be achieved ("kept live").

- Objective 1 states "what and how" has WHO PEN been implemented. I do not understand the what part here. Did you mean to write which of the PEN activities were implemented or the likes?

- Objective 2 states "outcomes and impact." I would define both (at least briefly) in the abstract for clarity.

- Given the timeline of this protocol review, I am wondering why your search will not extend beyond February 2025

- In the Introduction section, page 6, you have listed your specific questions. However, it appears that you have often lumped more than one question together. It is not clear to me what the justification is for doing so

-In the Methods section, page 12, you mention open inductive coding. Do you not see any scope of some deductive coding, given there's a lot of information on these results already?

- How will you move from coding to identifying themes and subthemes? The separation of these techniques is not apparent from your description, unfortunately

-Are your themes and subthemes already identified as implied by Table 3?

-"We will test this framework as an analytic pathway, which may provide us more in-depth understanding on the implementation process and outcomes across different stages and settings." What did you mean by "test" here?

-"Calculate risk ratios, mean differences, or changes from baseline, depending on the type of outcome." This part needs more detail. If you are not able to list all specific measures, you at least should mention as many as you can. Risk ratio or what?, for example.

All the best in your endeavour!

VERSION 1 - AUTHOR RESPONSE

Reviewer: Dr. Riffat Ara Shawon, University of Washington

Comments to the Author:

Dear Authors, thank you for taking on the task of compiling evidence on PEN intervention. Here are a few of my comments for your consideration.

- Abstract includes that this will be a "live" review. I am not clear from the abstract how it will be achieved ("kept live").

Many thanks for point this out. We will begin by producing an up-to-date baseline systematic review following the methods outlined in this protocol. Subsequently, updates will be conducted approximately every three years through the existing WHO Collaborating Centres and their network. Studies published after June 2025 will be incorporated in subsequent update. These updates will use the same protocol or a modified version, depending on the emergence of new evidence, and on policy or practice needs that require the most current data. Ultimately, the living evidence synthesis will inform future updates of the WHO PEN and implementing other related guidelines.

We add a subsection on the living systematic review approach under the method section (line 397-404).

We also modify the abstract (line 21-25).

- Objective 1 states "what and how" has WHO PEN been implemented. I do not understand the what part here. Did you mean to write which of the PEN activities were implemented or the likes?

Thank you for this helpful observation. In Objective 1, the phrase “what and how WHO PEN has been implemented” refers to two aspects of implementation. By “what,” we need to know which disease modules, intervention activities, elements or tools of WHO PEN have been implemented in different settings. By “how,” we need to examine the processes, approaches, strategies, targeted population, and contextual factors that shaped implementation.

We revise the ‘Implementation of WHO PEN and context of the review’ section to reflect this (line 117-119)

We also modify the abstract to improve clarity (line 15-20).

- Objective 2 states "outcomes and impact." I would define both (at least briefly) in the abstract for clarity.

Thank you for this useful suggestion. In Objective 2, “outcomes and impact” refer to implementation results. Outcomes means short to-medium term effects of WHO PEN implementation (e.g., changes health system readiness, provider performance, patient behavior or clinic changes). Impacts refer to more longer term effects on health system performance and population health.

We revise the ‘Implementation of WHO PEN and context of the review’ section to reflect this (line 120-121)

Similarly, we modify the abstract to improve clarity (line 15-20).

- Given the timeline of this protocol review, I am wondering why your search will not extend beyond February 2025

Thank you for raising this point. We revise cutoff in June 2025 reflects the timeline of the protocol development and initial pilot work of searching articles. Extending the search window further would risk delaying the review. However, because this review is designed as the foundation for a living systematic review, studies published after June 2025 will be captured in subsequent updates.

We add the following in the Living Systematic Review Approach section (line 400-401)

Studies published after June 2025 will be incorporated in subsequent updates.

- In the Introduction section, page 6, you have listed your specific questions. However, it appears that you have often lumped more than one question together. It is not clear to me what the justification is for doing so

Thank you for this thoughtful comment. You are correct that some of the listed questions combined more than one element, and we have revised the wording to improve clarity. We would also like to note that certain aspects of implementation are inherently interconnected and are best examined together to capture the complexity of the implementation process, rather than separating them into overly fragmented questions.

We edit and improve the listed specific questions (line 138-163)

We also added a question that was previously missing: What barriers and facilitators influence the implementation of WHO PEN? (line 150)

-In the Methods section, page 12, you mention open inductive coding. Do you not see any scope of some deductive coding, given there's a lot of information on these results already?

Thanks for raising an important point. Our initial plan was to conduct inductive coding to analyze qualitative data in the various contexts. However, we also agree that there is room for incorporating deductive coding, given barriers and facilitators of the implementation guided by implementation science framework such as Consolidated Framework for Implementation Research (CFIR). We revise the Methods section to clarify this blended approach. This blended approach will help ensure that the analysis captures both emergent insights and concepts already well-described in the literature.

We revise the Method section accordingly (line 317-321).

- How will you move from coding to identifying themes and subthemes? The separation of these techniques is not apparent from your description, unfortunately

Thank you for noting that this process was not sufficiently explicit. We will revise the description to clearly outline the analytic steps. Specifically, after initial coding, we add (line 324-331):

- Group related codes into preliminary categories.
- Develop themes and subthemes by identifying patterns, relationships, and overarching concepts, and map these to themes identified in existing systematic review where appropriate (see Table 3)
- Use CFIR framework to classify qualitative findings, applying both deductive (framework-based) and inductive (data-driven) approached.

-Are your themes and subthemes already identified as implied by Table 3?

Yes. Table 3 is intended to illustrate the themes and subthemes identified in the existing literature and implementation frameworks.

-"We will test this framework as an analytic pathway, which may provide us more in-depth understanding on the implementation process and outcomes across different stages and settings." What did you mean by "test" here?

Thank you for highlighting the ambiguity around the term “test.” Our intention was to indicate that we would apply the proposed four-phase framework to organize and interpret the included studies. To avoid confusion, we have revised the wording.

The revised text now reads (line 344-345):

“We will apply this framework as an analytical pathway to gain deeper insights into the implementation process and outcomes across various stages and settings.”

-"Calculate risk ratios, mean differences, or changes from baseline, depending on the type of outcome." This part needs more detail. If you are not able to list all specific measures, you at least should mention as many as you can. Risk ratio or what?, for example.

Thank you for highlighting the need for greater specificity. We revise the text as below to include some examples and clarify that the specific measures used are listed in Table 2 (line 365-367).

Calculate risk ratios (e.g., proportion of patients with controlled blood pressure or blood sugar), mean differences of continuous outcomes, or changes from baseline, depending on the type of outcome (Table 2).
